# Density but not climate affects the population growth rate of guanacos ( *Lama guanicoe*) (Artiodactyla, Camelidae)

**DOI:** 10.12688/f1000research.2-210.v3

**Published:** 2014-09-18

**Authors:** María Zubillaga, Oscar Skewes, Nicolás Soto, Jorge E Rabinovich

**Affiliations:** 1 Centro de Estudios Parasitológicos y de Vectores (CEPAVE, CONICET-CCT-La Plata, UNLP), Universidad Nacional de La Plata, La Plata, Argentina; 2 Universidad de Concepción, Chillán, Chile; 3 Servicio Agrícola Ganadero (SAG), XII Región, Chile

**Keywords:** Camelidae, climatic variables, density effects, guanaco, population regulation, population growth rate

## Abstract

We analyzed the effects of population density and climatic variables on the rate of population growth in the guanaco ( *Lama guanicoe*), a wild camelid species in South America. We used a time series of 36 years (1977-2012) of population sampling in Tierra del Fuego, Chile. Individuals were grouped in three age-classes: newborns, juveniles, and adults; for each year a female population transition matrix was constructed, and the population growth rate (λ) was estimated for each year as the matrix highest positive eigenvalue. We applied a regression analysis with finite population growth rate (λ) as dependent variable, and total guanaco population, sheep population, annual mean precipitation, and winter mean temperature as independent variables, with and without time lags. The effect of guanaco population size was statistically significant, but the effects of the sheep population and the climatic variables on guanaco population growth rate were not statistically significant.

## Introduction

In order to understand population dynamics and optimize the management of wildlife populations it is important to identify how populations are affected by environmental factors and their own density, particularly in species living in extreme environments. Mechanisms that regulate guanaco populations ( *Lama guanicoe*) are poorly known; this species was heavily exploited in the Patagonian steppe, and its numbers and distribution have diminished significantly during the last century because of grazing conflicts with a sheep-based society, and overhunting (the guanaco’s historical distribution has been reduced by 75% in Chile and Peru, and by 60% in Argentina) ^1^. The guanaco is in the Least Concern IUCN Red List category ( http://www.iucnredlist.org/, downloaded on 11 September 2013) but it has been included in Appendix II of CITES 2013 ( http://www.cites.org/eng/app/2013/E-Appendices-2013-06-12.pdf).

No population of any species can grow indefinitely, and population checks based upon different processes restrict population size and/or geographic distribution; these processes are either density-stabilizing or density-limiting ^2^. The former are of a biotic nature and depend on the interaction between individuals of the same or different species, while the latter are independent of population size. Stabilization results from density-dependence, with a regulatory effect that varies in intensity with the size or density of the population itself, however, not all density-dependent factors are density-stabilizing. The density-limiting factors can also be considered density-responsive because the *per capita* amount or availability of resources decreases as the population density increases. Thus, these two types of factors (density-stabilizing and density-limiting) rarely act independently: the density-limiting factors (generally of a physical and/or climatic nature), may determine the level at which populations become stabilized by the density-dependent processes, but they do not have a stabilizing capacity *per se*; for this reason many wildlife population dynamic and management models include the effects of climatic variables (e.g., Dennis & Otten, 2000; Colchero *et al.*, 2009) ^3, 4^.

The guanaco is one of the two extant wild South American camelids, and ranges from Northern Peru through Chile, and across Patagonia to southern Argentina and Chile, reaching Tierra del Fuego. It is found from sea level to nearly 4500 m on the Andes mountain range, and occupies a wide variety of habitats from hardpan deserts to scrublands to grasslands ^5^.

Although there are several studies on the population regulation processes in mammals in general ^6, 7^, and in ungulates in particular ^8, 11^, there are very few studies in wild South American camelids that analyze population growth regulation. There are several studies dealing with population dynamics of vicuña ( *Vicugna vicugna*) ^12– 14^ and guanacos ^15– 17^; however none of them considered the effect of density, livestock and climate on population growth rate.

Thus, our purpose in this work was to test the hypothesis that population density, sheep stock, and climatic variables, affect the growth rate of the guanaco population from the island of Tierra del Fuego, Chile.

## Materials and methods

### Study area

We analyzed the guanaco population of the “Cameron” ranch (-53.9 S, -69.3 W), with an area of the 2000 km ^2^ located in the Southern region of the Tierra del Fuego island, Chile. The altitude range is 0–300 m above sea level, and the ranch is a mosaic of steppe with fragments of forest; the latter is a deciduous forest *(Nothofagus spp)* while the steppe is composed of meadows, peats and prairies. The forested area is about 8.8% of the total study area, with some degree of forest clearance that offers adequate visibility for guanaco sampling. The guanaco is the dominant wild herbivore, while sheep are the dominant domestic species; densities of the latter have fluctuated in the last decades between 11 and 23 sheep/km ^2^ ^18^. In contrast to the mainland, the puma ( *Puma concolor*), the main predator of guanacos, is absent on the island; since 1977 guanaco hunting has been controlled by the Chilean National Forest Corporation (CONAF, according to its Spanish acronym) and the Chilean Agricultural and Livestock Service (SAG, according to its Spanish acronym).

The climate of the Tierra del Fuego island is characterized by an average annual precipitation of 200–400 mm, while in the forested areas the average precipitation fluctuates between 400–600 mm per year ^1^. The average annual temperature is 6.5°C and the average winter temperature is 2.2°C.

### Guanaco population sampling

Guanacos were counted for 34 consecutive years between 1977 and 2012 (except in 1986 and 1996), using the transect method with an undefined band width, from 1977 to 1995, and with a fixed width band from 1996 to 2012 (the latter with a maximum of 500 m to each side of the transect) ^20– 22^. The sampling methodology was changed by expert recommendation made in 1995 ^23^; in 2000 the fixed and the undefined band width methods were applied simultaneously, we tested both results and found no significant statistical difference between them. The sampling period was carried out in the autumn and lasted approximately 7 days between 10:30 and 19:00 h, with two observers in two 4×4 vehicles going over the main, secondary and local roads at speeds slower than 40 km/h. Despite randomly selected transects being recommended ^24^, preexisting roads were used because according to Soto (2010) ^18^ the existing system of roads is an adequate sample of the area. Each road was covered only once, and in addition to individual guanaco counts, the following were recorded: weather conditions, time, distance (km) from the starting point, GPS coordinates, observation distance from the transect (m) to guanacos, an estimate of the angle to the animal’s position, and – when the animals were observed in groups – the number of individuals, the type of social group, and its structure (sex, and the age class: newborn, juvenile and adult). Observation distances were made by naked eye. The road network and all geo-referenced observations were processed with the Arc View 9.3 Geographical Information System (GIS), and transferred using program Map Source ^®^. The cartography was kindly provided by the Chilean Agricultural and Livestock Service (SAG).

The population size was estimated as given in Soto ^18^ which was based on the King method modified by Leopold (1933) ^25^ described by Raedeke (1978) ^19^, given by:

$$N=\frac{\text{A}*\text{n}}{\text{2}*\text{y}*\text{x}}\;\;\;\;\;\text{(1)}$$

where *N* is the total population to be estimated, *A* is the total study area, *n* is the total number of animals counted, *x* is the total transect distance covered (m) rounded to one meter, and *y* is the average of the perpendicular distance (m) from the transect to the animals counted (the factor of 2 is included to consider that there is one band to each side of the transect). The variance ( *S ^2^*) was estimated by (2), with p = n/N, and used to estimate the 95% upper and lower confidence intervals.

$$S^{2}=\frac{\text{n}}{\text{p2}}*\frac{1-\text{p}\;\text{+}\;\text{n}}{\text{n}\;\text{+}\;\text{2}}\;\;\;\;\;(2)$$

Raedeke (1978) ^19^ claims that the Leopold method is valid when the following conditions are satisfied: (1) the road systems must be an adequate sampling of the study area, (2) the network road must be randomly distributed, and (3) the animals included in the sampling must be randomly distributed in relation to the observer’s route and he considers that in the south of the Tierra del Fuego Island these assumptions are fulfilled. Soto 2010 ^18^ compared population estimations by the Leopold and Distance methods and found that the value of the means estimate by Leopold methods fall within the confidence intervals estimated by Distance; additionally, as all sampling periods used the same methodology whatever bias might exist in the estimation of the mean abundance using the Leopold method, the relative changes among years (and thus the temporal trend) will be adequately represented; thus, we conclude that the Leopold’s method seems adequate for our purposes ^18^.

The area effectively surveyed in each sampling period was around 420 km ^2^ (about 20%, of the area under study).

### Climatic variables

We evaluated the possible impact of two climatic variables on guanaco population growth rate: average annual precipitation (mm/year) and winter temperature (as the average of the temperatures of June, July and August, in °C). These climatic variables were selected because some studies suggested that they have an influence on guanaco demographic parameters: Rey *et al.* 2012 ^26^ observed that after a severe drought the proportion of guanaco yearlings/females decreased significantly, and Sarno *et al.* 1999 ^17^ detected a negative effect of winter snowfall on guanaco juvenile survival; because we had insufficient snowfall data we used the winter temperature as a proxy for winter snowfall. We used the 25 years (1977–2002) precipitation and temperature time series of the CRU TS 2.1 database, compiled by the Tyndall Centre, Climatic Research Unit, School of Environmental Sciences of the University of East Anglia, United Kingdom ( http://www.cru.uea.ac.uk/cru/data/hrg.htm). As the CRU TS 2.1 data ended in 2002, we completed that time series for 2003 to 2012 from the closest meteorological station to the Cameron ranch: Punta Arenas (Chile); this data was downloaded from the Internet site of the Meteorological Service of Chile ( http://www.meteochile.gob.cl/).

### Sheep population

The livestock effect was evaluated by considering the sheep population, based on time series data obtained from Soto (personal communication). Only eight years of data were available (1980, 1985, 1990, 1995, 2000, 2005, 2008 and 2011); as the sheep population change between years was relatively smooth, we interpolated linearly between two consecutive data points to generate a complete time series of 36 years.

### Guanaco population analysis

We estimated the finite rate of increase (λ) as a measure of population growth rate for each of the 36 years of the guanaco data (population estimates were not available for years 1986 and 1996, and were linearly interpolated). To estimate λ we developed a three age-class (newborns, juveniles and adults) female-only matrix model, and estimated the matrix parameters by fitting the model predictions to the field total population estimates; we summed the three age classes and multiplied the result by two (guanacos have an approximately 50% sex-ratio). The fit was carried out in an Excel ^®^ spreadsheet using the Solver tool, and the sum of squares (SSQ) as goodness of fit criterion (details can be found in Rabinovich and Zubillaga, 2012 ^27^). This process resulted in a set of 36 population age-class structured matrices, and from each matrix we calculated the largest positive eigenvalue ^28^ as an estimate of λ, using the PopTools add-in, an Excel program developed by Greg Hood ( http://www.cse.csiro.au/poptools/).

### Population analysis

To test our hypothesis, we used a multiple regression analysis to check the relationship between the population growth rate (λ) as a dependent variable and guanaco population size (in natural logs), sheep population size (in natural logs), and climatic variables (annual mean precipitation in mm/year, and winter mean temperature in °C) as independent variables. In the case of the climatic variables we also evaluated the effects of various time lags (T), with T = 1 to 7 years for precipitation, and T = 1 year for winter temperature; the lags were applied by averaging the previous *T* lagged years, as suggested by Shaw *et al.* (2012) ^13^ for the vicuña population (these lagged variables were used in the regression analyses in addition to the non-lagged precipitation and winter temperature variables). The inclusion of time lags is convenient because it may detect possible cumulative climatic effects on ungulate population growth rates as has been observed in, for example, deer and moose ^29^ and in vicuñas ^13^. The multiple regression was carried out using the statistical package “ *lm*” of the *RStudio* software (Version 0.97.449 – ©2009–2012 RStudio, Inc.). Since for multiple regression analysis it is necessary to check that there is no collinearity (significant correlations among the predictor variables), we carried out a correlation analysis between independent variables, and used a *p* value < 0.05 to determine statistical significance. We then, for each of those variables that were significantly correlated among them, ran the same multiple regression analysis but replaced the correlated variables individually; i.e., we did as many multiple regression analyses as correlated variables were found. We used the Akaike index (AIC) as a goodness of fit criterion for model selection. We also carried out a simple linear regression analysis using λ as the dependent variable and the variable that was most statistically significant as the independent variable, in order to compare this result with the full regression model (guanaco population, sheep population and climatic variables as independent variables) and evaluate the advantage of incorporating the other variables in the analyses.

## Results

Data set 1 shows for each of the 36 years of guanaco sampling all the variables (dependent and independent) used in the regression analysis.

Update 1. Guanaco population abundance, sheep population abundance, estimated guanaco population annual rate of growth ( *λ*), average annual precipitation, and average winter temperature of the Cameron ranch, Tierra del Fuego Guanaco population abundance (individuals), lower and upper limits of the 95% confidence intervals (CI), sheep population abundance (individuals), estimated population annual rate of growth ( *λ*), average annual precipitation (P; mm/year), and average winter temperature (degrees Celsius). Guanaco population values were not available for years 1986 and 1996, and were linearly interpolated (using the values of the previous and following years); sheep population values were only available for years 1980, 1985, 1990, 1995, 2000, 2005, 2008 and 2011; and the missing years were also linearly interpolated.
Data has been updated in following columns: sheep population [ranges of changed values are only for years: 1981–1984; 1986–1989; 1991–1994; 1996–1999; 2001–2004; 2006–2007, 2009–2010]; data of winter temperature ( *Av winter temp*); annual precipitation ( *P*) [ranges of changed values are only for years: 2003–2011].

Correlation analysis between the climatic variables showed a statistically significant correlation ( *p* < 0.05) only among the precipitation variables for all the 1 to 7 year lags (but not with non-lagged precipitation), while the correlation results between all other independent climatic variables were not statistically significant. Thus, we ran seven regression analyses (each with each independent variables of lagged-precipitation). In all multiple regression analyses only the total population ( *Ln*Ntot) was statistically significant. We show the results of the regression with precipitation lagged T = 7 because is the multiple regression that resulted most statistically significant (AIC = -85.09 *p* < 0.017 and *F* = 3.154) ( Table 1).

**Table 1.** Results of multiple regression analysis between the population growth rate (λ) as dependent variable, and (in log scale) total guanaco population (LnNtot), sheep population (LnSheep), annual mean precipitation (Annual Precip), mean precipitation with lags of 7 years (Precip (T = 7)), winter mean temperature (Winter temp.), and winter mean temperature with lagged 1 year (Winter temp (T = 1)) as independent variables. The results show the regression coefficients (Estimate), their standard errors (Std. Error) and t-test values (t value), with their probability value ( *Pr(>|t|)*).

	Estimate	*Std. Error*	t value	*Pr(>|t|)*
Intercept	0.2536	1.2020	0.2110	0.8344
LnNtot	-0.0785	0.0238	-3.3050	0.0025
LnSheep	0.0972	0.0947	1.0260	0.3134
Annual Precip	-0.0001	0.0002	-0.3160	0.7546
Winter temp	0.0200	0.0174	1.1530	0.2584
Precip (T = 7)	0.0014	0.0008	1.7130	0.0974
Winter temp (T = 1)	0.0320	0.0174	1.8420	0.0758

The regression analysis, using λ as the dependent variable and only the guanaco population size (in natural logs, *LnNtot*) as the independent variable, was highly significant (AIC = -87.847, *p* < 0.0018, and *F* = 11.48,) and the resulting regression equation was λ = 1.6373 – 0.0552 (±0.0163) *Ln*Ntot ( Table 2).

**Table 2.** Results of the simple linear regression analysis between the population growth rate (λ) as dependent variable, and (in log scale) total guanaco population ( *LnNtot*). The interpretation of the statistical parameters are as in Table 1.

	Estimate	*Std. Error*	t value	*Pr(>|t|)*
Intercept	1.6373	0.1624	10.082	9.45E-12
LnNtot	-0.0552	0.0163	-3.388	0.00179

**Figure 1.**
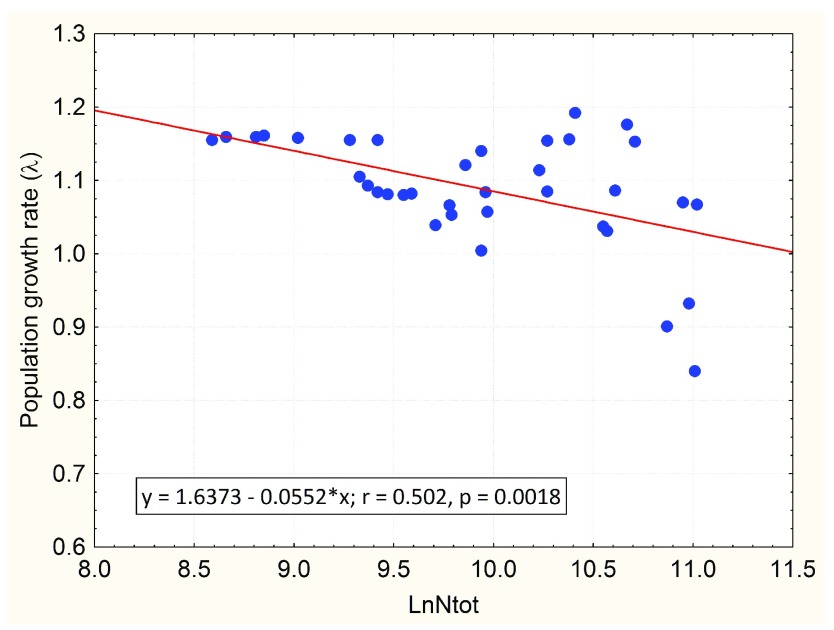
Effect of the guanaco population size on guanaco’s finite population growth rate (λ). On the x-axis the guanaco population from the Cameron ranch (Tierra del Fuego, Chile) was transformed to a natural logarithmic scale. The values of λ represent the finite population growth rate (on a per year time unit).

The negative slope of the regression equation is an indication of a density-dependent process, since when the population increases the *per capita* population growth rate decreases ( Figure 1). The intercept of the regression line at *λ* = 1 (population at equilibrium) results in a population size of 103,000 guanacos (51 guanaco/km ^2^), which can be considered as an estimate of the carrying capacity of the Cameron ranch for this guanaco population.

## Discussion

We were not able to confirm the expected effect of either climatic variables or sheep population on the population rate of growth (λ) in the guanaco population of the Cameron ranch. We expected an effect of climatic variables because Sarno *et al.* (1999) ^17^ found a relationship between winter weather and guanaco yearling’s survival, and Rey *et al.* (2012) ^26^ recorded a negative effect of a severe drought on guanaco recruitment. Thus we anticipated that as winter temperature and/or annual precipitation decrease, the guanaco population growth rate might also decrease, as shown by Mech *et al.* 1987 ^29^ with deer and moose. They also used a simple linear regression, and found that as winter snow accumulation increased, annual percentage change in population numbers decreased. With respect to sheep population, we also expected to find some type of relationship because this domestic herbivore is considered a competitor of the guanaco ^30^ for food and water. Our negative results are even more surprising because in the last 10–12 years of the time-series data the guanaco population fluctuated remarkably, suggesting some extraneous factor in addition to between population density, maybe climatic variables. It is well known that environmental effects are more important when the population size is near the carrying capacity, particularly in large mammals characterized by low reproductive rates, long life-spans, and populations that are resource-limited (features typical of species referred to as the “ *K-selected*” species) ^31^. The difference between our results and those of Sarno *et al.* (1999) ^17^ may reflect that these authors used the average winter snowfall while we considered average winter temperature.

The results of the guanaco population sampling suggest a certain trend in the population size, with a more or less exponential growth in the first few years, becoming more variable as the population grows, with more marked fluctuations during the last 10–12 years; this may imply that the population is becoming stabilized, possibly approaching its carrying capacity. On the other hand, the sampling results of those last 10–12 years show abrupt “jumps” in some years (for example 2004–2005) that may be the consequence of the mobility of guanacos from the Cameron ranch to neighboring sites and vice versa; since the transect sampling does not identify individuals the effects of possible local displacements could not be considered.

On the other hand the lack of effects of the sheep population size on the finite population growth rate (λ) in this guanaco population conforms well with a recent study ^32^ in Southern Chile that found that the potential competition between guanaco and sheep is low.

In contrast to our results, a study by Taper & Gogan (2002) ^33^ on the Yellowstone elk population, with weather variables included as covariates within a dynamic model, found that spring precipitation had a positive regression coefficient.

Contrary to what was expected based on the literature of ungulate population dynamics, weather variables do not seem to influence the density-dependent population growth rate of the Cameron ranch guanaco population. Our aim was to carry out a preliminary analysis that would help identify some of the regulatory processes in this guanaco population and so we used a simple multiple regression analysis as a first step in that direction. However, we note that in order to test the effects of climatic variables on population regulation of large ungulates such as the guanaco, the use of a population dynamic model would be recommended. A population dynamic model would better account for the interaction between density-dependent processes and weather variables than a simple multiple regression between the latter and guanaco population growth rate.

## Data availability

*F1000Research*: Dataset 1. Update 1. Guanaco population abundance, sheep population abundance, estimated population annual rate of growth, average annual precipitation, and average winter temperature of the Cameron ranch, Tierra del Fuego, 10.5256/f1000research.2375.d35439 ^34^
