# Supplementary material for: Density but not climate affects the population growth rate of guanacos ( Lama guanicoe) (Artiodactyla, Camelidae)
Source: F1000Res. 2014 Sep 18;2:210. Originally published 2013 Oct 9. [Version 3] doi: 10.12688/f1000research.2-210.v3 (PMC4149246; doi:10.12688/f1000research.2-210.v3)
Supplement: Peer review history

# Peer review: Referee response for version 2

**DOI:** 10.5256/f1000research.4501.r5906

**Published:** 2014-08-21

**Authors:** Pablo Acebes

**Affiliations:** 1 Department of Ecology, Autonomous University of Madrid, Madrid, Spain

The authors have improved the ms from their first submitted version. Please see the comments below to further improve the article:

- First paragraph (introduction): please put guanaco in the singular.
- Guanaco population sampling section: please replace ‘SIG’ with ‘GIS’.
- Guanaco population sampling section: please remove ‘surface’ in “A is the total study area surface”.
- Results: part of the results is a justification of the analyses carried out and should be placed in Material and Methods.
- Discussion: in my view one of the most important results is the absence of negative effects of sheep on guanaco population growth rate that indeed has been cited as one of the major drivers of guanaco population decline particularly in Patagonia in the 20 century. For that reason I think authors should discuss this important result more deeply.

I have read this submission. I believe that I have an appropriate level of expertise to confirm that it is of an acceptable scientific standard.

# Peer review: Referee response for version 2

**DOI:** 10.5256/f1000research.4501.r2049

**Published:** 2014-08-04

**Authors:** Gary Luck

**Affiliations:** 1 Institute for Land, Water, and Society, Charles Sturt University, Albury, Australia

I have cross checked the comments from the two original referees with the responses from the authors and consider that the authors have adequately addressed all comments. The study has various limitations, such as a limited set of predictor variables, lack of the use of a sampling technique (e.g. Distance sampling) that accounts for differences in detectability, and application of a simple regression analysis. The authors have adequately addressed each of these major issues in their response to referees - though readers should be aware of these limitations when interpreting the results of the study.

I have read this submission. I believe that I have an appropriate level of expertise to confirm that it is of an acceptable scientific standard.

# Peer review: Referee response for version 1

**DOI:** 10.5256/f1000research.2588.r2847

**Published:** 2013-12-19

**Authors:** Pablo Acebes

**Affiliations:** 1 Department of Ecology, Autonomous University of Madrid, Madrid, Spain

The authors explore the population dynamics of a long-term monitored guanaco population (36 years) in the Chilean Tierra del Fuego, trying to discern if the process is governed by population size and/or environmental (climatic) variables, assuming that guanaco growth rate follows a density-dependent process. Although the topic is very interesting, the authors have room to improve the manuscript. Below the authors will find major and minor comments.

**Major comments**

The manuscript is classified as a “Research article” when indeed it seems a “Short note”. It would greatly benefit from deeper and more detailed explanations of:

1. The theory of ungulate population dynamics, driven by density-dependent and density-independent processes; please expand this topic in the introduction and discuss more deeply your results considering the theoretical framework (see comments below).
2. The census methodology (see comments below).
3. Statistical modeling  (see comments below).

Ungulate populations are regulated via endogenous forces (density-dependent processes), and exogenous forces (density-independent): weather and predation. Weather determines the primary production, i.e. the resource (forage) availability, which in turn determines the carrying capacity. On the other hand weather, through extreme conditions (e.g. severe winter climate), modulates population dynamics (starvation, survival). Predation is considered an important force which shapes population dynamics as well. In addition, poaching or overhunting act as predation, and competition with other ungulates is another important exogenous force. With this framework authors should justify:

1. The background supporting the study, as it currently is a bit poor, in my view. The authors should explain more in detail, for example, which are the differences between the density-stabilizing and the density-limiting processes, or the evidence of including climatic variables when modelling population growth rate trends.
2. The use of these climatic variables: the average annual precipitation and winter temperature. Without any justification, authors could have also included others variables in the models, as climatic variables do not account for the pattern described, but this doesn’t necessarily mean that climate hasn’t got any effect on the guanaco population growth rate. Moreover authors should explain if these two climatic variables are selected as indicators of primary production and harsh weather conditions.
3. As authors stated in introduction, guanaco populations have undergone overhunting (poaching) and competition with sheep, these being two of the major forces of their dramatic decline in the last century.  For that reason, it is quite surprising that no variables related to livestock were included in the modelling procedure. The population has increased a lot in the studied period (x8) and just climate and/or density-dependent process do not explain alone such increase in my view. Therefore authors should consider including livestock effects, its influence on resource availability along the studied period and the mentioned overhunting, as this could really explain the detected trend.

The guanaco sampling method has changed during the studied period (different band widths). Besides, were the transects chosen based on the topography and vegetation cover of the ranch to ensure that all areas were visible to census-takers along their transects? I am not sure if that could be a source of error, but authors should discuss this point. Moreover, I recommend authors to re-analyze their data using the DISTANCE program (Buckland *et al.,* 2004) to estimate population size over the period, as the analyses performed by this package seems much more robust that the one employed by authors. If they do so, authors could also include topographic variables in the models to see whether the resulting models change. The reason is that the authors do not explain if guanacos occur in both biomes (steppe and forest), because if that was true, detection of guanacos was completely different in both ecosystems. Therefore the band width of 500 or 1000m is not valid for forests.

Regarding the population size estimated along the 36 years (Supplementary table), important (huge) differences are found, for example between 2004 and 2005 or between 2006 and 2007, or between 2007 and 2008. My concern is if such “jumps” could be related to the census surveyed. Please discuss this point.

The analytical approach employed by the authors doesn’t seem appropriate in my view, as they also recognize in the discussion, and that could be the reason for not finding any climatic variable significant in the model:

1. The authors use a stepwise regression when other approaches such as logistic regressions, Ricker or Beverton–Holt models could give more suitable results. Please explain or justify your decision.
2. The selection of the best model among the candidates should be done by Akaike’s information.

**Minor comments**

1. Refer to climatic variables or climatic effects, instead of climatic covariables.
2. Explain more in detail Corani & Gatto’s studies.
3. Please remove information related to metabolic studies between guanacos and sheep (1st paragraph of study area section).
4. Please add *Nothofagus* spp among the brackets after deciduous forest (study area section) if it is the case.
5. Give more information about livestock ranching, density, and size and the relation of livestock rangers with guanacos along the studied period (study area section).
6. Are the guanacos found both in the steppe and the forest biomes?
7. Please re-write the paragraph related to guanaco sampling through transect method in the consecutive periods (it is not clear enough).
8. Please remove information related to binoculars and GPS brands (did the authors use the same gadgets across 36 years?).
9. Explain the interest of using time lags (T) as covariables in the model.
10. Please add the Statistica version employed.
11. Please move explanations of the analyses perform from results to material & methods section.

I have read this submission. I believe that I have an appropriate level of expertise to confirm that it is of an acceptable scientific standard, however I have significant reservations, as outlined above.

# Response

**Published:** 2014-05-14

**Authors:** Maria Zubillaga

**Affiliations:** CONICET-UNLP, Argentina

- *"The background supporting the study, as it currently is a bit poor, in my view. The authors should explain more in detail, for example, which are the differences between the density-stabilizing and the density-limiting processes, or the evidence of including climatic variables when modelling population growth rate trends."*
  After the following paragraph that was already in the original text:

*“No population of any species can grow indefinitely, and population checks based upon different processes restrict population size and/or geographic distribution; these processes are either density-stabilizing or density-limiting”*

We added the following paragraph explaining the differences between the density-stabilizing and the density-limiting processes.

*“The former are of a biotic nature and depends on the interaction between individuals of the same or different species, while the latter are independent of population size. Stabilization results from density-dependence, with a regulatory effect that varies in intensity with the size or density of the population itself, however, not all density-dependent factors are density-stabilizing. The density-limiting factors can also be considered density-responsive because the per capita amount or availability of resources decreases as the population density increases. Thus, these two types of factors (density-stabilizing and density-limiting) rarely act independently: the density-limiting factors (generally of a physical and/or climatic nature), may determine the level at which populations become stabilized by the density-dependent processes, but they do not have a stabilizing capacity per se; for this reason many wildlife population dynamic and management models include the effects of climatic variables (e.g., Dennis & Otten, 2000; Colchero et al., 2009) ^3,4^”.*

 

- *"The use of these climatic variables: the average annual precipitation and winter temperature. Without any justification, authors could have also included others variables in the models, as climatic variables do not account for the pattern described, but this doesn’t necessarily mean that climate hasn’t got any effect on the guanaco population growth rate. Moreover authors should explain if these two climatic variables are selected as indicators of primary production and harsh weather conditions."*
  To explain and justify the use of these climatic variables the paragraph below was incorporated in the section “Climatic variables”:

“ *These climatic variables were selected because some studies suggested that they have influence on guanaco demographic parameters: Rey et al. 2012 ^26^* *observed that after a severe drought the proportion guanaco yearlings/female decreased significantly, and Sarno et al. 1999 ^17^* *detected a negative effect of winter snowfall on guanaco juvenile survival; because we had insufficient snowfall data we used the winter temperature as a proxy for winter snowfall.”*

 

- *"As authors stated in introduction, guanaco populations have undergone overhunting (poaching) and competition with sheep, these being two of the major forces of their dramatic decline in the last century.  For that reason, it is quite surprising that no variables related to livestock were included in the modelling procedure. The population has increased a lot in the studied period (x8) and just climate and/or density-dependent process do not explain alone such increase in my view. Therefore authors should consider including livestock effects, its influence on resource availability along the studied period and the mentioned overhunting, as this could really explain the detected trend."*
  In relation with the “poaching” the following paragraph was incorporated in section “Study area” to justify why this variable was not considered in our study:
*“In contrast to the continent, the puma (Puma concolor), the main predator of guanacos, is absent in the island; since 1977 the guanaco hunting has been controlled by Chilean National Forest Corporation (CONAF, according to its Spanish acronym) and the Chilean Agricultural and Livestock Service (SAG, according to its Spanish acronym).”*
  ​In relation to the suggestion to incorporate livestock in the analysis, we re-analyzed our multiple regression incorporating the variable livestock as sheep population. We re-processed the regressions incorporating the sheep population as a new independent variable as a possible predictor of the guanaco population growth rate (l); so our new analysis increased from 5 to 6 independent variables: sheep population, guanaco population size, winter temperature, winter temperature with a lag of 1 year, annual precipitation, annual precipitation with a lag of 3 years. Our main results didn’t change and it was confirmed that only the guanaco population size seems to have an effect on the population growth rate (l). These changes can be found in the “Methods” and in the “Results” sections.
- *“The guanaco sampling method has changed during the studied period (different band widths). Besides, were the transects chosen based on the topography and vegetation cover of the ranch to ensure that all areas were visible to census-takers along their transects? I am not sure if that could be a source of error, but authors should discuss this point.”*
  In the Section “Guanaco population sampling” we added the following paragraphs where we explain the motive of the change and justify the transects chosen:

 

*“The sampling methodology was changed by an expert recommendation made in 1995* *^23^* *; as in year 2000 the fixed and the undefined band width methods were applied simultaneously, we tested both results and found no significant statistical difference between them.”*

*“Despite randomly selected transects are recommended* *^24^* *, preexisting roads were used because according to Soto (2010)* *^18^* *the existing system of roads is an adequate sample of the area.”*

 

- “ *Moreover, I recommend authors to re-analyze their data using the DISTANCE program (Buckland et al., 2004) to estimate population size over the period, as the analyses performed by this package seems much more robust that the one employed by authors. If they do so, authors could also include topographic variables in the models to see whether the resulting models change. The reason is that the authors do not explain if guanacos occur in both biomes (steppe and forest), because if that was true, detection of guanacos was completely different in both ecosystems. Therefore the band width of 500 or 1000m is not valid for forests.”*
   In the Section “Guanaco population sampling” the following paragraphs was incorporated to justify the methodology used:
*“Raedeke (1978)* *^19^* *claims that the Leopold method is valid when the following conditions are satisfied: (1) the road systems must be an adequate sampling of the study area, (2) the network road must be randomly distributed, and (3) the animals included in the sampling must be randomly distributed in relation to the observer’s route; and he considers that in the south of the Tierra del Fuego Island these assumptions are fulfilled. Soto 2010* *^18^* *compared population estimations by the Leopold and Distance methods and found that the value of the means estimate by Leopold methods fall within the confidence intervals estimated by Distance; additionally, as all sampling periods used the same methodology whatever bias might exist in the estimation of the mean abundance using the Leopold method, the relative changes among years (and thus the temporal trend) will be adequately represented; thus, we conclude that the Leopold’s method seems adequate for our purposes* *^18^* *.”*
  In relation to the forest and the sampling methodology, as the area occupied by forests is low and it has some degree of intervention, the visibility of the guanaco is adequate, hence we considered that the forest is a minor component and that does not affect the efficiency of the sampling procedure. In the section “Study area” the next paragraph was incorporated:
*“The forested area is about 8.8% of the total study area, with different degrees of forest clearance that offers adequate visibility for guanaco sampling.”*

 

- *“Regarding the population size estimated along the 36 years (Supplementary table), important (huge) differences are found, for example between 2004 and 2005 or between 2006 and 2007, or between 2007 and 2008. My concern is if such “jumps” could be related to the census surveyed. Please discuss this point.”*
  In the “Discussion” section we added the following discussion as required by the reviewer:
*“The results of the guanaco population sampling suggest a certain trend in the population size, with a sort of exponential growth in the first few years, becoming more variable as the population grows, with more marked fluctuations during the last 10-12 years; this may imply that the population is becoming stabilized, possibly approaching its carrying capacity. On the other hand, the sampling results of those last 10-12 years show abrupt “jumps” in some years (for example 2004-2005) that may be the consequence of the mobility of guanacos from the Cameron ranch to neighboring sites and vice versa; since the transect sampling does not identify individuals the effects of possible local displacements could not be considered.”*
- *“The authors used a stepwise regression when other approaches such as logistic regressions, Ricker or Beverton-Holt models could give more suitable results. Please explain or justify your decision.”*
  Our goal was to carry out a preliminary analysis that would guide us in the study of the population regulation process in the guanaco. As there are studies in ungulate that have used simple multiple regression analysis and obtained good/satisfactory results, we considered that it was of interest to test this technique as a first step in such an analysis.
  In the manuscript the following paragraph was incorporated in the “Discussion” section to justify our decision: 
*"Contrary to what was expected based on the bibliography of ungulate’s population dynamics, weather variables do not seem to influence the density-dependent population growth rate process of the guanaco population of the Cameron ranch. Our aim was to carry out a preliminary analysis that would help identify some of the regulation processes in guanaco population; so we used a simple multiple regression analysis as a first step in that direction; nevertheless, our conclusion is that in order to test the effects of climatic variables  on population regulation of large ungulates as the guanaco, the use of a population dynamic model would be recommended, for they seem to account better  for the interaction between density-dependent processes and weather variables than a simple regression between the latter and guanaco population growth rate.”*
- *“The selection of the best model among the candidates should be done by Akaike’s information* *”*
  This suggestion was considered and the respective Akaike’s values were calculated.

**Minor comments:**

1. *“Refer to climatic variables or climatic effects, instead of climatic covariables.”*
  The recommendation was applied.
2. *“Explain more in detail Corani & Gatto’s studies.”*
  With the previous changes we realize that the citation of Corani and Gatto (2007) is not necessary any more. Mainly because the essence of that paper is the proposal of a methodology that is not strictly relevant to our work. The phrase with this citation was deleted.
3. *“Please remove information related to metabolic studies between guanacos and sheep (1st paragraph of study area section).”*
   The suggestion was carried out.
*  *
4. *“Please add Nothofagus spp* *among the brackets after deciduous forest (study area section) if it is the case.”*
  This information is already incorporated.
5. *“Give more information about livestock ranching, density, and size and the relation of livestock rangers with guanacos along the studied period (study area section).”*
  This information was already incorporate in the section “Sheep population”
6. *“Are the guanacos found both in the steppe and the forest biomes?” *
  Yes, the guanacos use both biomes, but they mostly use the open habitat (i.e. steppes and meadows).  
7. *“Please re-write the paragraph related to guanaco sampling through transect method in the consecutive periods (it is not clear enough).”*
  The paragraph was improved.
8. *“Please remove information related to binoculars and GPS brands (did the authors use the same gadgets across 36 years?).”*
  The recommendation was carried out.
9. “Explain the interest of using time lags (T) as covariables in the model.”
  The next paragraph was incorporated in section “Population analysis”:
  “ *The inclusion of time lags is convenient because it may detect possible cumulative climatic effects on ungulate population growth rates as it was observed in, e.g., deer and moose* *^29^* *and in vicuñas ^13^”.*
10. *“Please add the Statistica version employed.”*
  The *Statistica* tools was replaced by language ***R*** (Version 0.97.449 – ©2009-2012 RStudio, Inc.) to obtain the value of Akaike and satisfy the item 3.2 (see above).
11. *“Please move explanations of the analyses perform from results to material & methods section.”*
  The recommendation was carried out.

# Peer review: Referee response for version 1

**DOI:** 10.5256/f1000research.2588.r2048

**Published:** 2013-11-20

**Authors:** Ricardo Baldi

**Affiliations:** 1 Terrestrial Ecology Research Unit, Centro Nacional Patagónico, Puerto Madyrn, Argentina

The authors hypothesized that population density and climatic variables affect the growth rate of the population of guanacos in the study area. However, as it is detailed under the “Study area” subsection, the domestic sheep share the area with the guanaco and substantial changes in stocking rate have been reported for the ranch during the study period. It is known that sheep and guanacos select similar diets across different parts of the range they share, and usually sheep presence and abundance explain a substantial amount of variation in the abundance of guanacos, included the study area comprising the Estancia Cameron as described by Raedeke (1979). In his work, Raedeke described that sheep are managed “rotationally” around the property, taken seasonally to different areas in winter and summer, and this makes guanacos move seasonally to the forest habitat. Therefore, it would be expected that variations in sheep abundance, not only seasonally but in total numbers through time, could affect the estimates of guanaco abundance. In my opinion, this is a major issue in the design of this work. Also, as explained below, the survey design of this work could play a key role in the quality of the estimates obtained, which is reflected also in the interpretation of results. All of these do not mean that density dependent processes are not likely to occur, but to my understanding it is not possible to rule out other processes to be conclusive.

**Guanaco population sampling**

The surveys were conducted at a 2,000 km ^2^ ranch where the habitat is a mosaic of steppe and forest biomes. The authors indicate that they surveyed guanacos using the “transect method with a variable width” from 1977 to 2000. Is this the distance sampling? If so, it is based on measuring the perpendicular distance from the transect to the object. Also, it is says that the surveying methodology was changed to “fixed width band” in 2001 and subsequent years. Some comments and questions on these issues regarding survey design are:

1. Were both the steppe and forest habitats surveyed using the same methodology? Transect lines to observe individuals and make direct counts are commonly used in open habitats, but not in the forest. However if this was not the case, the authors need to account for the variation in the probability of detection as it is expected to vary significantly between such contrasting habitat types.
2. There was a change in the surveying method, which also brings the point of detection probability. While the line transect method assumes that the probability of detecting objects on the line (distance=0) is p=1, and as objects are far from the line the probability of detection follows a certain declining function, the main assumption of the strip transect method is that the probability of detecting objects within the strip is 1 (i.e. no objects are left undetected). This variation in the probability of detection according to the methodology applied during each period must be discussed. Also, the ways that the density estimates were obtained are unclear and must be specified (i.e. distance sampling, other calculation methods).
3. Regarding the estimate of population size, several considerations can be made about the extrapolation of absolute densities to population numbers. First, the spatial pattern of the transects surveyed could be determinant of biased estimates, if the objects are not randomly distributed in relation to the survey line (i.e. if the transect follows a geographic feature in such a way the animals are either attracted or deterred). Perhaps the transects are located across certain areas – for example the steppe – but not the forest and thus the estimate could be biased towards the density calculated for the surveyed portion of the total area. This is especially important at the time of extrapolate densities to obtain population size numbers, as densities are strictly valid for the areas effectively surveyed while traveling along the transect lines. In any case, it would be useful to provide the distribution of surveyed lines across the area. The second point I find relevant to discuss regarding survey design is the proportion of the area effectively surveyed. How many km ^2^ were comprised by the lines and strips surveyed? Again, the extrapolation of densities to population size at a larger scale must account for possible limitations related to the survey design and effort invested.

**Results and discussion**

Major fluctuations of the guanaco population occurred during the last 10-12 years analyzed, as can be found in the additional data file provided, and also pointed out by the authors. But this variation could well be due to the changes in methodology at the time of the population surveys. As said above, it is necessary to provide detailed information on the type of survey conducted, the differences with the previous method and the way the density estimates were obtained. It would be informative if the authors provide the errors associated with the population estimates.

As proposed above, sheep densities could be affecting guanaco population trends, but the design of the work does not allow accounting for this factor. In my opinion, sheep densities must be included as an explanatory variable as there is evidence they affect guanaco numbers. Also, as the authors concluded that in this study the guanaco populations was near carrying capacity, and this must be common to both guanacos and sheep, thus sheep must be considered in the analysis as its density was markedly variable during the study period. Another factor that should be considered at the time of the discussion is the possibility of guanacos moving from the surveyed surroundings or even from Cameron to neighboring sites, as they may respond to changes in the spatial availability of resources by occupying different sites. In summary, the marked fluctuations in population size require the examination and discussion of other possible factors before concluding that density dependent processes would drive population trends.

I have read this submission. I believe that I have an appropriate level of expertise to confirm that it is of an acceptable scientific standard, however I have significant reservations, as outlined above.

# Response

**Published:** 2014-05-14

**Authors:** Maria Zubillaga

**Affiliations:** CONICET-UNLP, Argentina

- *"The authors hypothesized that population density and climatic variables affect the growth rate of the population of guanacos in the study area. However, as it is detailed under the “Study area” subsection, the domestic sheep share the area with the guanaco and substantial changes in stocking rate have been reported for the ranch during the study period. It is known that sheep and guanacos select similar diets across different parts of the range they share, and usually sheep presence and abundance explain a substantial amount of variation in the abundance of guanacos, included the study area comprising the Estancia Cameron as described by Raedeke (1979). In his work, Raedeke described that sheep are managed “rotationally” around the property, taken seasonally to different areas in winter and summer, and this makes guanacos move seasonally to the forest habitat. Therefore, it would be expected that variations in sheep abundance, not only seasonally but in total numbers through time, could affect the estimates of guanaco abundance. In my opinion, this is a major issue in the design of this work. Also, as explained below, the survey design of this work could play a key role in the quality of the estimates obtained, which is reflected also in the interpretation of results. All of these do not mean that density dependent processes are not likely to occur, but to my understanding it is not possible to rule out other processes to be conclusive."* 
  In relation to the suggestion to incorporate livestock in the analysis, we re-analyzed our regression incorporating the variable livestock as sheep population; we re-processed the regressions incorporating the sheep population as a new independent variable of the guanaco population growth rate (l); so our new analysis increased from 5 to 6 independent variables: sheep population, guanaco population size, winter temperature, winter temperature with a lag of 1 year, annual precipitation, annual precipitation with a lag of 3 years. Our main results didn’t change and it was confirmed that only the guanaco population size seems to have an effect on the population growth rate (l). These changes can be found in the “Methods” and in the “Results” paragraphs. 
- *"The surveys were conducted at a 2,000 km ^2^ ranch where the habitat is a mosaic of steppe and forest biomes. The authors indicate that they surveyed guanacos using the “transect method with a variable width” from 1977 to 2000. Is this the distance sampling? If so, it is based on measuring the perpendicular distance from the transect to the object."*
  No, the sampling methodology did not use the program Distance. The citations of Buckland et al. (1993 and 2001) were removed to avoid possible misinterpretations with the sampling methodology used.The method used to estimate the population size was the King method modified by Leopold (1933) as described in Raedeke (1978). In the manuscript it was explained and justified this method with the following paragraph:

*“Raedeke (1978) ^19^ claims that the Leopold method is valid when the following conditions are satisfied: (1) the road systems must be an adequate sampling of the study area, (2) the network road must be randomly distributed, and (3) the animals included in the sampling must be randomly distributed in relation to the observer’s route; and he considers that in the south of the Tierra del Fuego Island these assumptions are fulfilled. Soto 2010 ^18^ compared population estimations by the Leopold and Distance methods and found that the value of the means estimate by Leopold methods fall within the confidence intervals estimated by Distance; additionally, as all sampling periods used the same methodology whatever bias might exist in the estimation of the mean abundance using the Leopold method, the relative changes among years (and thus the temporal trend) will be adequately represented; thus, we conclude that the Leopold’s method seems adequate for our purposes ^18^. The area effectively surveyed in each sampling period was around 420 km² (about 20%, of the area under study).”*

- *"Also, it is says that the surveying methodology was changed to “fixed width band” in 2001 and subsequent years. Some comments and questions on these issues regarding survey design are:*

1. *Were both the steppe and forest habitats surveyed using the same methodology? Transect lines to observe individuals and make direct counts are commonly used in open habitats, but not in the forest. However if this was not the case, the authors need to account for the variation in the probability of detection as it is expected to vary significantly between such contrasting habitat types."*
  We corrected in the manuscript the years where the change in band width occurred: variable band width from 1977 to 1995, and with a fixed width band (with a maximum of 500 m to each side of the transect) from 1996 to 2012.
  The area occupied by forest is low and it has some degree of intervention, the visibility of the guanaco is adequate, hence we considered that the forest is a minor component and it does not affect the efficiency of sampling. In the section “Study area” the following paragraph was incorporated:

*"The forested area is about 8.8% of the total study area, with different degrees of forest clearance that offers adequate visibility for guanaco sampling.”*

 

- *"There was a change in the surveying method, which also brings the point of detection probability. While the line transect method assumes that the probability of detecting objects on the line (distance=0) is p=1, and as objects are far from the line the probability of detection follows a certain declining function, the main assumption of the strip transect method is that the probability of detecting objects within the strip is 1 (i.e. no objects are left undetected). This variation in the probability of detection according to the methodology applied during each period must be discussed. Also, the ways that the density estimates were obtained are unclear and must be specified (i.e. distance sampling, other calculation methods)."*
  We improved this section and the next paragraph was incorporate to explain this change. ​The population size was estimated by King method modified by Leopold (1933) as described by Raedeke (1978), which was also clarified in the manuscript.

*"The sampling methodology was changed by an expert recommendation made in 1995 ^23^; as in year 2000 the fixed and the undefined band width methods were applied simultaneously, we tested both results and found no significant statistical difference between them.”*

 

- *"Regarding the estimate of population size, several considerations can be made about the extrapolation of absolute densities to population numbers. First, the spatial pattern of the transects surveyed could be determinant of biased estimates, if the objects are not randomly distributed in relation to the survey line (i.e. if the transect follows a geographic feature in such a way the animals are either attracted or deterred). Perhaps the transects are located across certain areas – for example the steppe – but not the forest and thus the estimate could be biased towards the density calculated for the surveyed portion of the total area. This is especially important at the time of extrapolate densities to obtain population size numbers, as densities are strictly valid for the areas effectively surveyed while traveling along the transect lines. In any case, it would be useful to provide the distribution of surveyed lines across the area. The second point I find relevant to discuss regarding survey design is the proportion of the area effectively surveyed. How many km ^2^ were comprised by the lines and strips surveyed? Again, the extrapolation of densities to population size at a larger scale must account for possible limitations related to the survey design and effort invested."*
  Although the transects are not randomly distributed, the preexisting roads can be considered as an adequate sample of the area. This comment was incorporate in the manuscript.

*"Despite randomly selected transects are recommended ^24^, preexisting roads were used because according to Soto (2010) ^18^ the existing system of roads is an adequate sample of the area.”*

- The second point was considered and the area effectively surveyed was estimated. In the “Guanaco population sampling” section the following paragraph was incorporated:

*"The area effectively surveyed in each sampling period was around 420 km² (about 20%, of the area under study).”*

 

- *"Major fluctuations of the guanaco population occurred during the last 10-12 years analyzed, as can be found in the additional data file provided, and also pointed out by the authors. But this variation could well be due to the changes in methodology at the time of the population surveys. As said above, it is necessary to provide detailed information on the type of survey conducted, the differences with the previous method and the way the density estimates were obtained. It would be informative if the authors provide the errors associated with the population estimates."*
  The errors associated with the population estimates are given in Table S1 where the lower and upper limits of the 95% confidence intervals (CI) are shown.
- *"As proposed above, sheep densities could be affecting guanaco population trends, but the design of the work does not allow accounting for this factor. In my opinion, sheep densities must be included as an explanatory variable as there is evidence they affect guanaco numbers. Also, as the authors concluded that in this study the guanaco populations was near carrying capacity, and this must be common to both guanacos and sheep, thus sheep must be considered in the analysis as its density was markedly variable during the study period. Another factor that should be considered at the time of the discussion is the possibility of guanacos moving from the surveyed surroundings or even from Cameron to neighboring sites, as they may respond to changes in the spatial availability of resources by occupying different sites. In summary, the marked fluctuations in population size require the examination and discussion of other possible factors before concluding that density dependent processes would drive population trends."*
  This factor (sheep population) has been incorporated, and the corresponding analysis was carried out; see in the “Materials and methods” section “Sheep Population” and the section “Results”.​ In the “Discussion” section the following paragraph was included where we recognized the possibility of guanaco moving as possible source of error in the sampling:

*“The results of the guanaco population sampling suggest a certain trend in the population size, with a sort of exponential growth in the first few years, becoming more variable as the population grows, with more marked fluctuations during the last 10-12 years; this may imply that the population is becoming stabilized, possibly approaching its carrying capacity. On the other hand, the sampling results of those last 10-12 years show abrupt “jumps” in some years (for example 2004-2005) that may be the consequence of the mobility of guanacos from the Cameron ranch to neighboring sites and vice versa; since the transect sampling does not identify individuals the effects of possible local displacements could not be considered.”*
